# Spike developmental stages and ABA role in spikelet primordia abortion contribute to the final yield in barley (*Hordeum vulgare* L.)

**DOI:** 10.1186/s40529-019-0261-2

**Published:** 2019-07-10

**Authors:** Faiza Boussora, Mohamed Allam, Ferdaous Guasmi, Ali Ferchichi, Twan Rutten, Mats Hansson, Helmy M. Youssef, Andreas Börner

**Affiliations:** 1 Leibniz Institute of Plant Genetics and Crop Plant Research (IPK), Corrensstraße 3, 06466 Gatersleben, Germany; 2 Faculty of Agriculture, Cairo University, Giza, 12613 Egypt; 3 Institute of Arid Lands of Medenine, Route du Djorf Km 22.5, Médénine, Tunisia; 4 Faculty of Sciences of Bizerte (FSB), 7021 Zarzouna, Bizerte, Tunisia; 5 Institut National Agronomique de Tunis, 43 Avenue Charles Nicolle, 1082 Tunis, Tunisia; 6 Department of Biology, Lund University, Sölvegatan 35, 22362 Lund, Sweden; 7 Institute of Agriculture, Lithuanian Research Centre for Agriculture and Forestry, Akademija, Lithuania

**Keywords:** Barley, Salinity, ABA, Spike development, Primordia abortion, Spikelet/floret abortion

## Abstract

**Background:**

Salinity is a significant environmental stress factor limiting crops productivity. Barley (*Hordeum vulgare* L.) has a natural tolerance to salinity stress, making it an interesting study object in stress biology research. In the present study, for the first time the effect of salinity stress on barley inflorescence developmental stages was investigated. Five spring barley genotypes irrigated with saline water (12.5 ds/m NaCl) were compared to controls treated with normal tap water. We measured abscisic acid (ABA) concentrations in the apical, central and basal sections of the immature inflorescence at green anther (GA) stage. The role of ABA in spikelet primordia development, atrophy and abortion and final yield was evaluated.

**Results:**

A time course experiment starting from double ridge until green anther (GA) stages revealed that salinity reduced the length of spike developmental stages in all genotypes causing shortened of the plant life cycle. The shortened plant life cycle negatively affected plant height and number of tillers/plant. Salinity also affected spikelet primordia development. In both control and salinity treated plants apical spikelet abortion started in late awn primordium (AP) stage. However, under salinity treatment, significantly more spikelets were aborted, thus directly affecting plant yield potential. ABA, which plays a role in the spikelet/floret abortion process, was markedly elevated in the base and apex of salt treated spikes correlating with an increased spikelet abortion in these regions.

**Conclusions:**

Overall, salinity treatment reduced all plant and yield-related parameters investigated and turned some of the correlations among them from positive to negative or vice versa. Investigations of ABA role in floral development and phase duration of barley spike showed that, ABA regulates the spikelet/floret abortion process affecting the yield potential under salinity and control conditions.

**Electronic supplementary material:**

The online version of this article (10.1186/s40529-019-0261-2) contains supplementary material, which is available to authorized users.

## Background

Abiotic stresses due to salinity occurs naturally (Dai 2011) but has become a growing global problem due to human activities such as salt mining (Ghassemi et al. 1995) and poor irrigation systems (Marcum and Pessarakli 2006). At present more than 800 million hectares of agricultural land are affected by salinity and/or sodicity stress (Munns 2005; Farooq et al. 2015). In arid and semiarid countries, agricultural production is limited by water availability and the resources of water are insufficient for the growing human population. As fresh water is allocated in priority for drinking purposes, irrigation water is often of poor quality. Nowadays, it has been estimated that about 20% of the cultivated land worldwide is affected by salinity (Jamil et al. 2011). The corresponding proportion of irrigated agricultural land is 33%, expected to reach 50% of the arable land by the year 2050 (Jamil et al. 2011). The growing salinity problem in arid and semi-arid regions needs an urgent solution where research aiming to understand the effects of salinity on cereals production should be combined with genetic efforts to develop salt tolerant crops for the future of agriculture (Shannon 1984; Owens 2001; Kausar et al. 2013). Biochemical pathways, morphological and physiological processes including seed germination, growth and development are affected by salinity (Willenborg et al. 2004) causing yield and quality reduction (Basalah 2010; Bagues et al. 2018). However, plant species differ in their response to salinity stress (Torech and Thompson 1993; Sarabi et al. 2017).

**Fig. 1:**
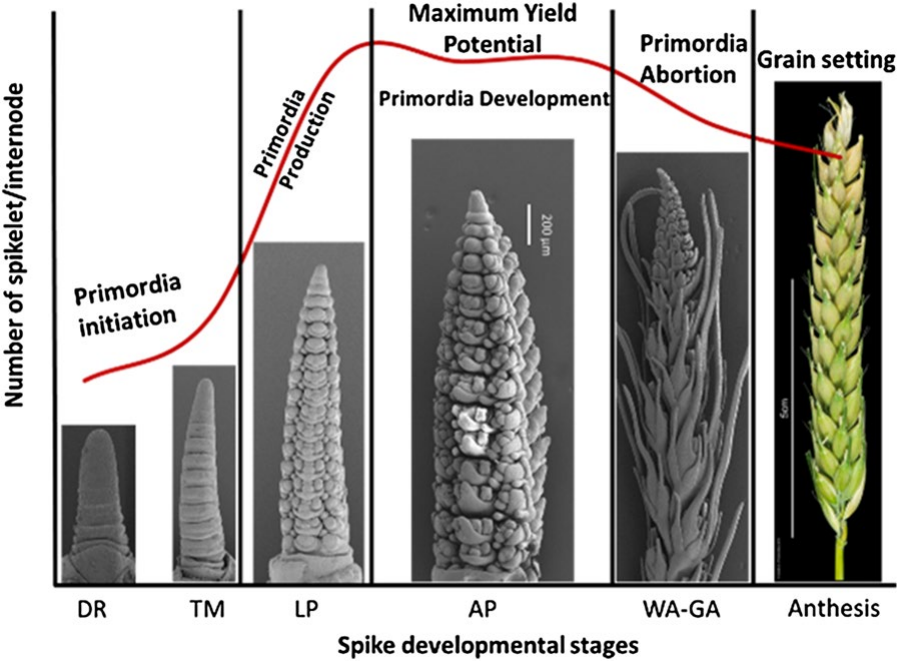
The spikelet primordia initiation, growth and development, abortion and grain setting phases in barley. *DR* double ridge, *TM* triple mound, *LP* lemma primordium, *AP* awn primordium, *WA* white anther, *GA* green anther

Barley (*Hordeum vulgare* L.) is one of the oldest cereal crops known to be cultivated since about 10,000 years. It has a natural tolerance to drought and salinity stresses. The inflorescence of cultivated barley is an indeterminate spike that produces three single-flowered spikelets at each rachis internode that make it unique among the grasses. Barley row type varies from two-rowed to six-rowed and is controlled by at least five independent mutant loci that include *six*-*rowed*-*spike1* (*vrs1*), *vrs2*, *vrs3*, *vrs4,* and *Intermedium*-*c* (*Int*-*c*). These loci are located on barley chromosomes 2HL, 5HL, 1HS, 3HS and 4HS, respectively (Pourkheirandish and Komatsuda 2007). The yield production is controlled by number of tillers (side shoots) and number of spikelets per spike and both are negatively correlated to each other. Spike developmental and growth stages (Fig. 1) play a very important role in defining the seed yield. Therefore, improved grain yield through enhanced spikelet survival is a key objective of many cereals breeding programs (Alqudah and Schnurbusch 2014). The maximum yield potential per spike is represented by the number of spikelets per spike at the awn primordium stage (Riggs and Kirby 1978; Waddington et al. 1983; Kirby and Appleyard 1987; Kernich et al. 1997; Alqudah and Schnurbusch 2014).

At the awn primordium stage six-rowed barley displays more spikelets/florets primordia per spike than two-rowed barley (Whingwiri and Stern 1982; Kirby and Appleyard 1987; Kernich et al. 1997; Miralles et al. 2000; del Moral et al. 2002; Arisnabarreta and Miralles 2006). Inflorescence development and growth, phase duration and organ patterning are influenced by phytohormones such as auxin (IAA), cytokinin (CK) and abscisic acid (ABA) (Su et al. 2011; Matsoukas 2014; Youssef et al. 2017). ABA also is the primary hormone that mediates plant responses to stress such as drought and salinity (Wu et al. 1997; Wilkinson and Davies 2010; Lee and Luan 2012). It has been suggested that the ABA level correlates with the plant resistance to stress (Maslenkova et al. 1993; Lee and Luan 2012) including salinity stress (Nilsen and Orcutt 1996; Suzuki et al. 2016). ABA is supposedly involved in the induction of the synthesis of the 26-kDa protein Osmotin which accumulation depends on the presence of NaCl (Bressan et al. 1985). ABA may also influence stomatal conductivity affecting tissue hydraulics (Collins and Kerrigan 1974; Davies and Zhang 1991; Freundl et al. 2000; Hose et al. 2002; Du et al. 2013) growth in response to drought or salinity through changes in cell wall extensibility (Bacon 1999; Cramer et al. 1998; Dodd and Davies 1996; Thompson et al. 1997) and apoplastic pH in plants (Bacon et al. 1998). Other studies have focused on the effect of ABA accumulation and a decrease in IAA and CK on the progression of senescence in salinized plant organs (Albacete et al. 2008; Ghanem et al. 2008). Salinity affects both vegetative and reproductive developmental stages and reduces shoot growth and the number of florets per ear. Furthermore, salinity increases sterility and changes the time of flowering and maturity in grasses (Läuchli and Epstein 1990). In the present work we studied the response of inflorescence developmental stages, spikelet primordia development, and ABA concentrations in the inflorescence of five spring barley genotypes to salinity stress and related this to final yield.

## Materials and methods

### Plant material

Five six-rowed spring barley genotypes, Ardhaoui, Kounouz, Lemsi, Manel and Rihane, maintained at the Tunisian gene bank were selected for this study based on their importance and yield under stress conditions (Table 1).

**Table 1:** Names, year of release and characteristics of the barley genotypes studied (Chaabane et al. 2009; Ben Ghanem and El Felah 2011; Ben Youssef et al. 2011)

Name	Year of release	Characteristics	Yield (t/ha)
Ardhaoui	Local Tunisian variety	Adapted to the driest regions	≈ 4.5
Kounouz	Selected by INRAT in 2011	Resistance to fungal diseases	≈ 5.2
Lemsi	Selected by INRAT in 2009	Used as fodder	≈ 4.0
Manel	Selected by INRAT in 1996	Suitable for wetlands	≈ 5.5
Rihane	Introduced from ICARDA in 1982	Adapted to semi-arid regions	≈ 5.0

*INRAT* Institut National de Recherche Agronomique de Tunis, *ICARDA* International Center for Agriculture Research in the Dry Areas

### Growing conditions and salinity treatment

The five barley genotypes were grown in IPK-Gatersleben greenhouse under long day conditions 16 h/8 h (day/night) and temperature of ~ 20 ± 2 °C during the day and ~ 16 ± 2 °C during the night. Per genotype two sets of 96 seeds were germinated in 96 wells plates, one watered with tap water as control and the second treated with saline water (12.5 ds/m NaCl, selected based on preliminary where the plants died at more than 12.5 ds/m NaCl concentration) starting from the day of planting. When seedlings reached three leaves stage, they were transferred into 14 cm diameter pots, irrigated with either tap water or saline water. Agricultural practices were performed as recommended, including pest, disease and weed control.

### Plant and spike phenotyping

To establish spike developmental stage, every second day after transfer into 14 cm pots, two plants of each genotype were dissected under a stereomicroscope (Stemi 2000-c, Carl Zeiss Micro Imaging GmbH, Gottingen, Germany). To compare spike developmental under salinity stress with control conditions, spikes of five or more plants at the developmental stages: Double Ridge (DR), Triple Mound (TM), Glum Primordia (GP), Stamen Primordium (SP), Lemma Primordium, Awn Primordium (AP), White Anthers (WA) and Green Anthers (GA) according to Kirby and Appleyard (1987), were collected for electron micrographs. After anthesis ten control and ten salinity treated plants were scored for plant height, number of tillers and main spike length. After complete maturity all spikes from each plant were collected for yield. Yield-related parameters seed length, seed width, seed area, number of seeds/plant, plant seeds weight and 1000-Grain weight) were measured. The experiment was repeated three times in the same green house under the same conditions.

### Scanning electron microscopy (SEM)

For SEM analysis, isolated barley spikes were fixed with 4% formaldehyde in 50 mM phosphate buffer, pH 7.0 for 16 h. After dehydration in a graded ethanol series and critical point drying in a Bal-Tec critical point dryer (Bal-Tec AG, Balzers, Switzerland), spikes were gold sputtered in an Edwards S150B sputter coater (Edwards High Vacuum Inc., Crowley, West Sussex, UK) and examined in a Hitachi S-4100 SEM (Hisco Europe, Ratingen, Germany) at 5 kV acceleration voltage. Digital recordings were made and stored as Tiff-image files.

### ABA determination

To compare ABA concentration along the developing spike, of each genotype 12 to 16 spikes at GA stages were collected and sectioned into basal, central, and apical parts (Youssef et al. 2017). After freeze-drying 20 to 50 mg dry weight was used to extract ABA according to Kojima et al. (2009) and Seo et al. (2011). ABA analysis by GC/MS (Shimadzu GC 2010 A chromatograph) was performed as described in Okamoto et al. (2009). Data from four biological replicates were analyzed and significance values were calculated.

### Statistical analysis

The experiment was arranged as a completely randomized design with ten replicates per genotype per treatment (control and salinity). Main effects of genotypes, control and salinity treatments, along with the corresponding interactions were tested using two-way analysis of variance (ANOVA). Significance of differences between means was estimated with Tukey’s HSD (Honest Significant Difference test). Pearson correlation coefficients for pairwise comparisons between all traits were computed. All statistical analyses in this study were conducted using R 3.5.3 (R Core Team 2018).

## Results

### Effect of salinity treatment on plant height and number of tillers

**Fig. 2:**
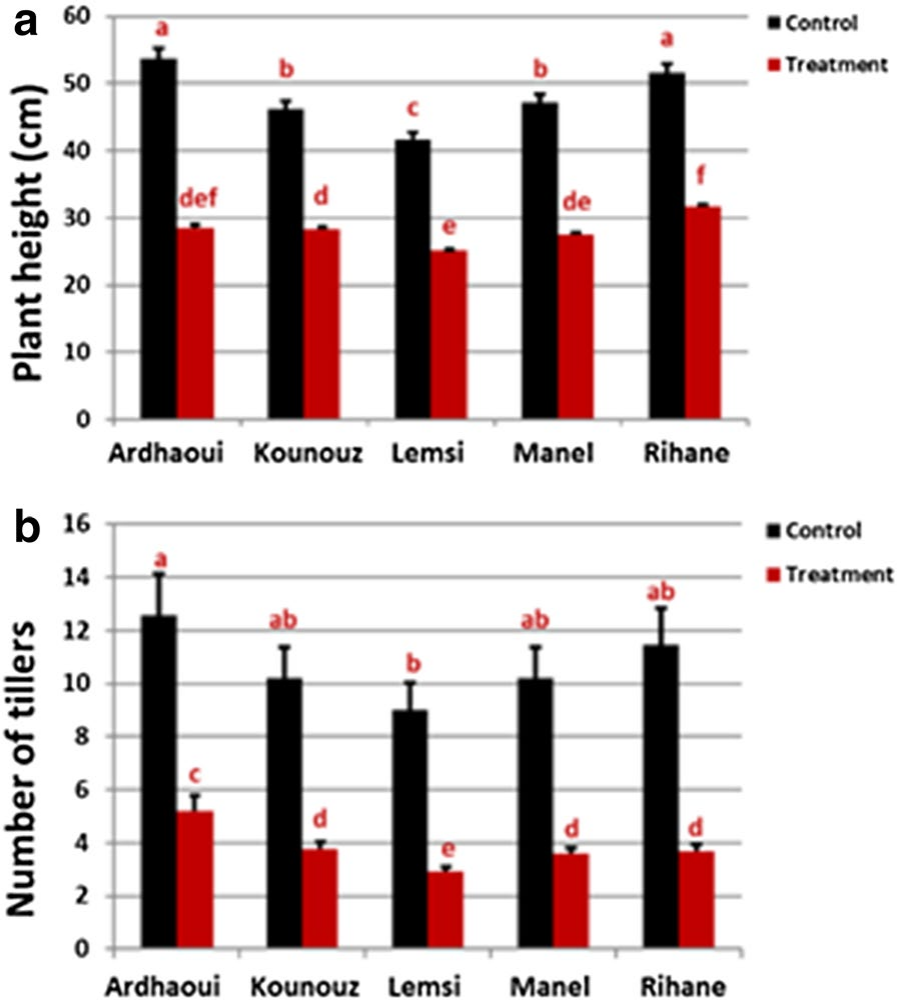
Plant height (**a**) and number of tillers (**b**) for the five barley genotypes under control and salinity treatment conditions. Significant differences among the genotypes, treatments and the interaction showed with the red letters and has been calculated using R 3.5.3 (*P* value < 0.05) (color figure online)

Five six-rowed spring barley genotypes, Ardhaoui, Kounouz, Lemsi, Manel and Rihane, were grown with or without salt. Even under control conditions the barley genotypes tested differed significantly with regard to plant height and number of tillers (Fig. 2). Plant height and number of tillers/plant were highest in the genotypes Ardhaoui and Rihane. These values were significantly smaller in the other three genotypes with the by far lowest values found in the genotype Lemsi (Fig. 2). The salinity treatment caused a substantial reduction in both plant height and number of tillers in all genotypes (Fig. 2, Additional file 1: Fig. S1). Under salinity plant height was largest in the genotype Rihane and lowest in the genotype Lemsi. For number of tillers/plant highest values were found in the genotype Ardhaoui, lowest again in the genotype Lemsi (Fig. 2).

### Salinity affects the length of spike developmental stages

**Fig. 3:**
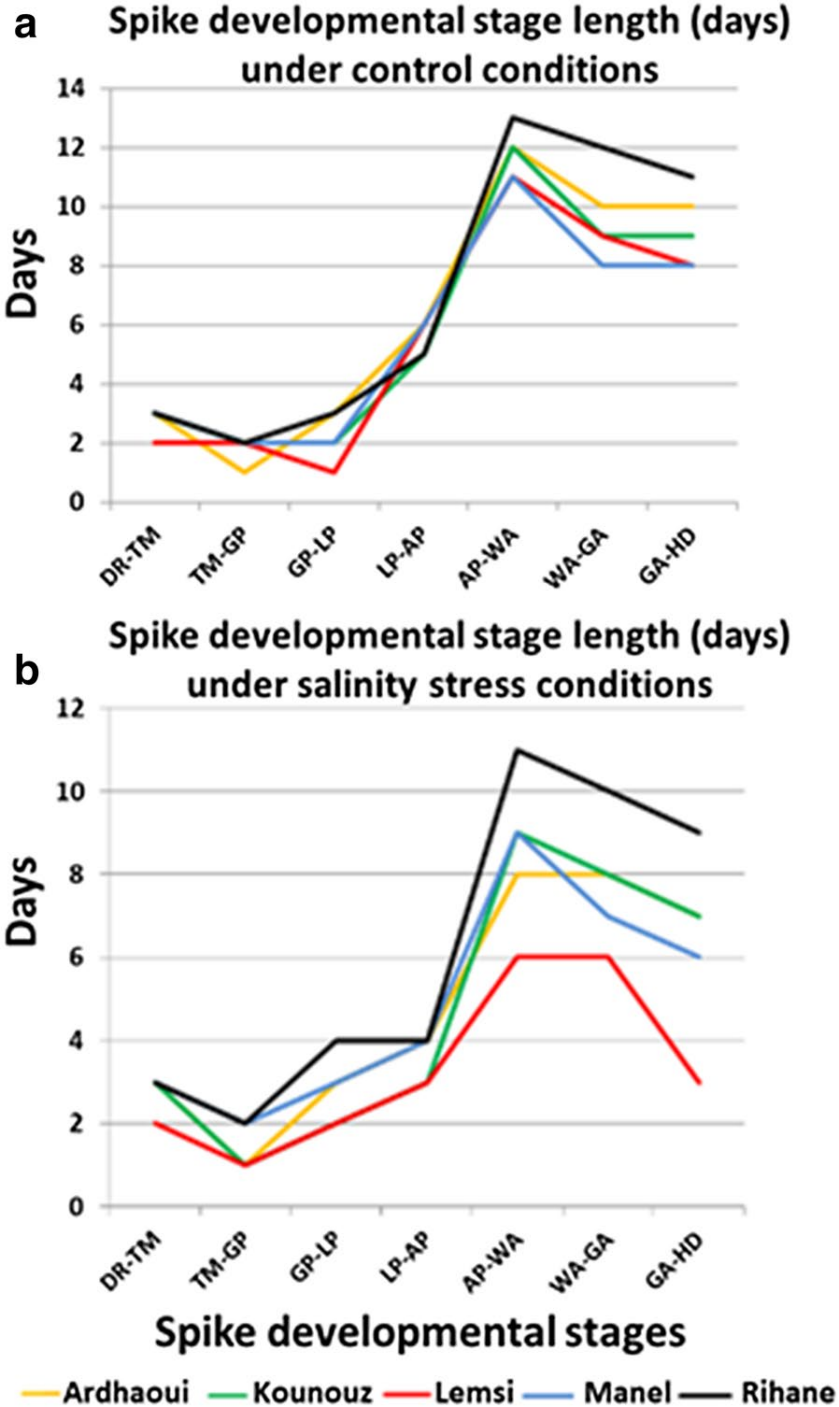
Spike developmental stage length (Days) under control conditions (**a**) and salinity stress conditions (**b**). *DR* double ridge, TM triple mound, *GP* glume primordia, *LP* lemma primordium, *AP* awn primordium, *WA* white anther, *GA* green anther, *HD* heading

The duration from planting to each of the eight developmental stages were measured for the five barley genotypes. When grown under control conditions, no differences were found between the genotypes from the double ridge (DR) stage to/through the white anther (WA) stage except for the Lemsi genotype (Fig. 3a). Lemsi not only developed faster but also reached flowering (heading day, HD) earlier than the four other genotypes (Additional file 2: Table S1). The other four genotypes started to diverge from each other after the WA stage. While genotype Manel reached green anther (GA) and heading (HD) stage at 44 and 52 days after planting (DAP), respectively, these values were 45 and 54 DAP for the genotype Kounouz, 48 and 58 DAP for the genotype Ardhaoui, and 51 and 62 DAP for the genotype Rihane (Fig. 3a and Additional file 2: Table S1). Stronger differences between the genotypes were seen under salinity treatment. Starting from Lemma Primordium (LP) stage, all genotypes reached subsequent spike developmental stages faster under salinity treatment than under control conditions (Fig. 3b). The shortest stage lengths for LP-AP, AP-WA, WA-GA and GA-HD were recorded for the Lemsi genotype taking 3, 6, 6 and 3 days, respectively (Fig. 3b). Consequently the Lemsi genotype was also the earliest to reach heading (33 DAP). The genotypes Ardhaoui, Kounouz and Manel reached heading at 45, 43 and 44 DAP, respectively. With LP-AP, AP-WA, WA-GA and GA-HD lasted 4, 11, 10 and 9 days, respectively (Fig. 3b), stage length reduction compared to control conditions was least severe in genotype Rihane which was the last to reach heading at 54 DAP.

### Salinity reduces the yield potential

**Fig. 4:**
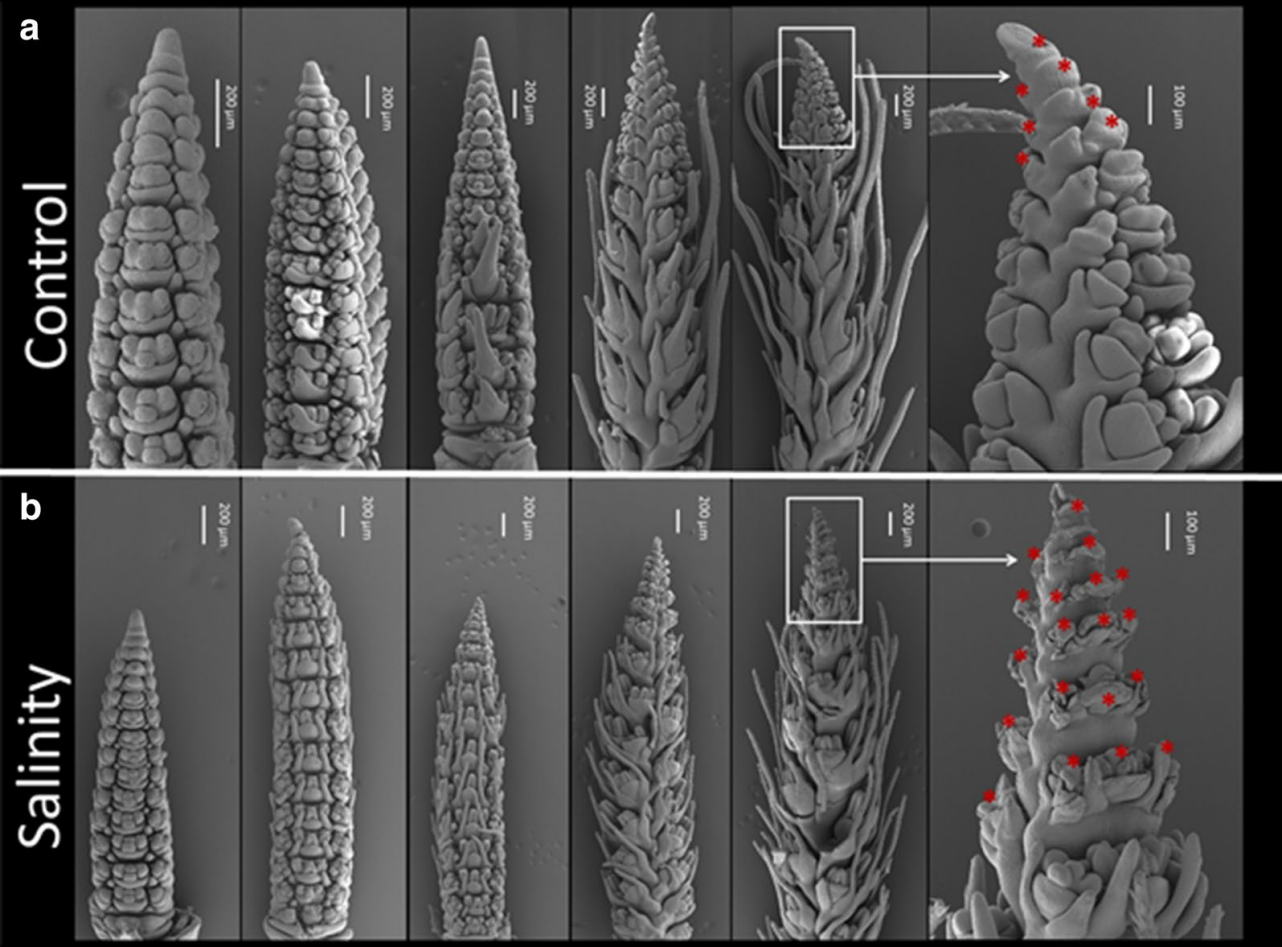
Barley spike development under control (**a**) and salinity treatment (**b**) conditions. Red stars showed the undeveloped/later-aborted spikelets (color figure online)

Scanning electron micrographs from DR to GA showed a clear effect of salinity on the spikelet primordia development. In the early stages from DR to AP, no obvious difference between the control and salinity conditions was found. In both control and salinity treated plants an atrophy of the basal and apical spikelets became evident from late AP stage onwards (red stars in Fig. 4). The number of aborted spikelets, however, was significantly higher under salinity treatment thus directly affecting plant yield potential. Spikelet abortion was more substantial in the apical part than in the basal part of the spike (Fig. 4).

### Salinity affects ABA concentration in the immature spike

**Fig. 5:**
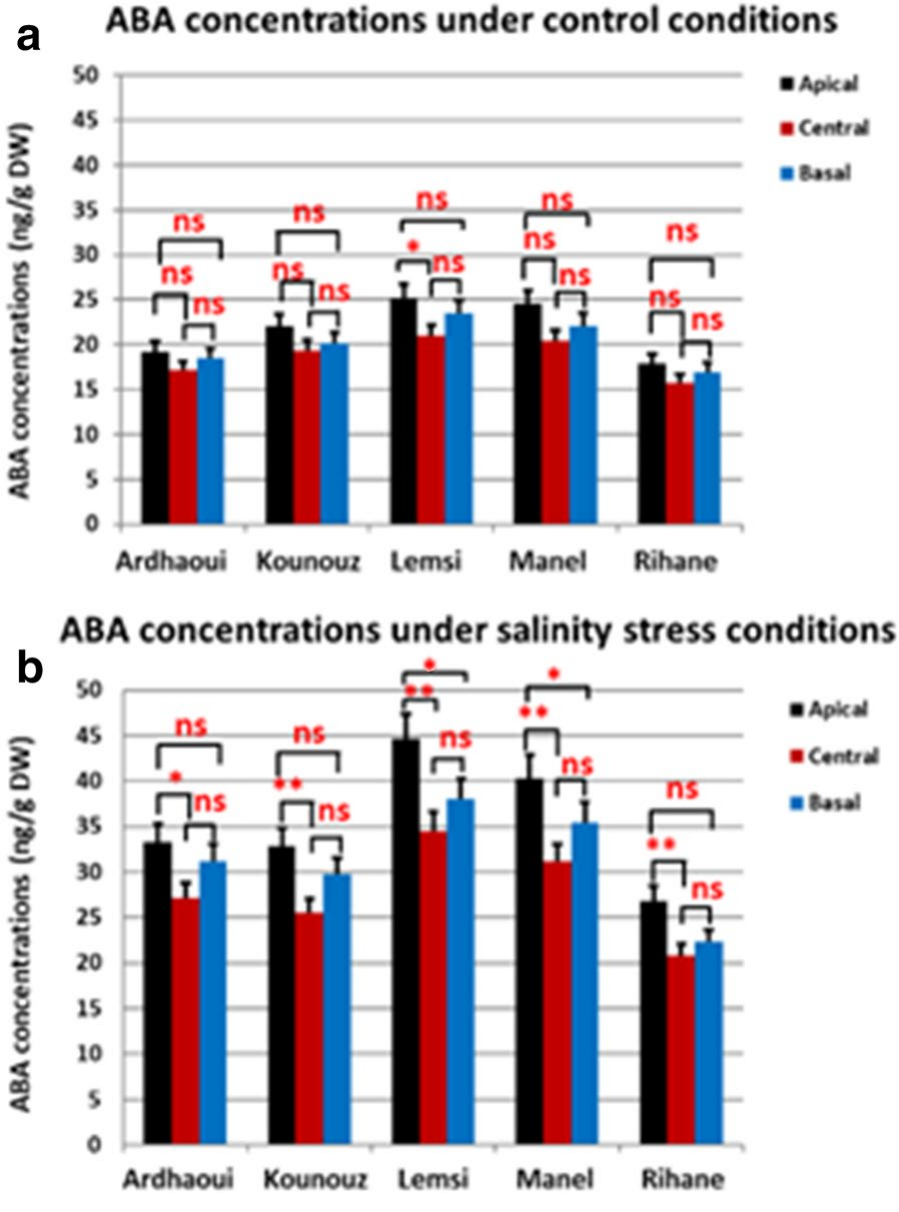
ABA concentrations (ng/g DW) in apical, central and basal spike sections at green anther stage of the five spring barley genotypes under control (**a**) and salinity stress (**b**) conditions. *Significant (*P* < 0.05%). **High significant (*P* < 0.01). ‘ns’ 0 non-significant. Significant differences among the genotypes and the spike sections has been calculated using R 3.5.3

Reports of local ABA accumulation in response to salinity stress (Wu et al. 1997; Suzuki et al. 2016) and observed spikelet atrophy at the apical and the basal sections of the spike at the GA stage (Fig. 4), called for a study on the differences of ABA concentrations along the spike at this stage. The results revealed that salinity caused an overall increase in the ABA concentrations within the spike. As under control conditions, however, ABA were always highest in the apical section and lowest in the central section of the spike. Among the barley genotypes ABA concentration under both control and salinity condition was highest in the salinity sensitive genotypes Lemsi and Manel (Fig. 5). The enhanced concentration of ABA and a concurrent increase in spikelet abortion under salinity (Fig. 4b) thus underlines the role of ABA in abortion phenomena.

### Effect of salinity treatment on yield traits

**Fig. 6:**
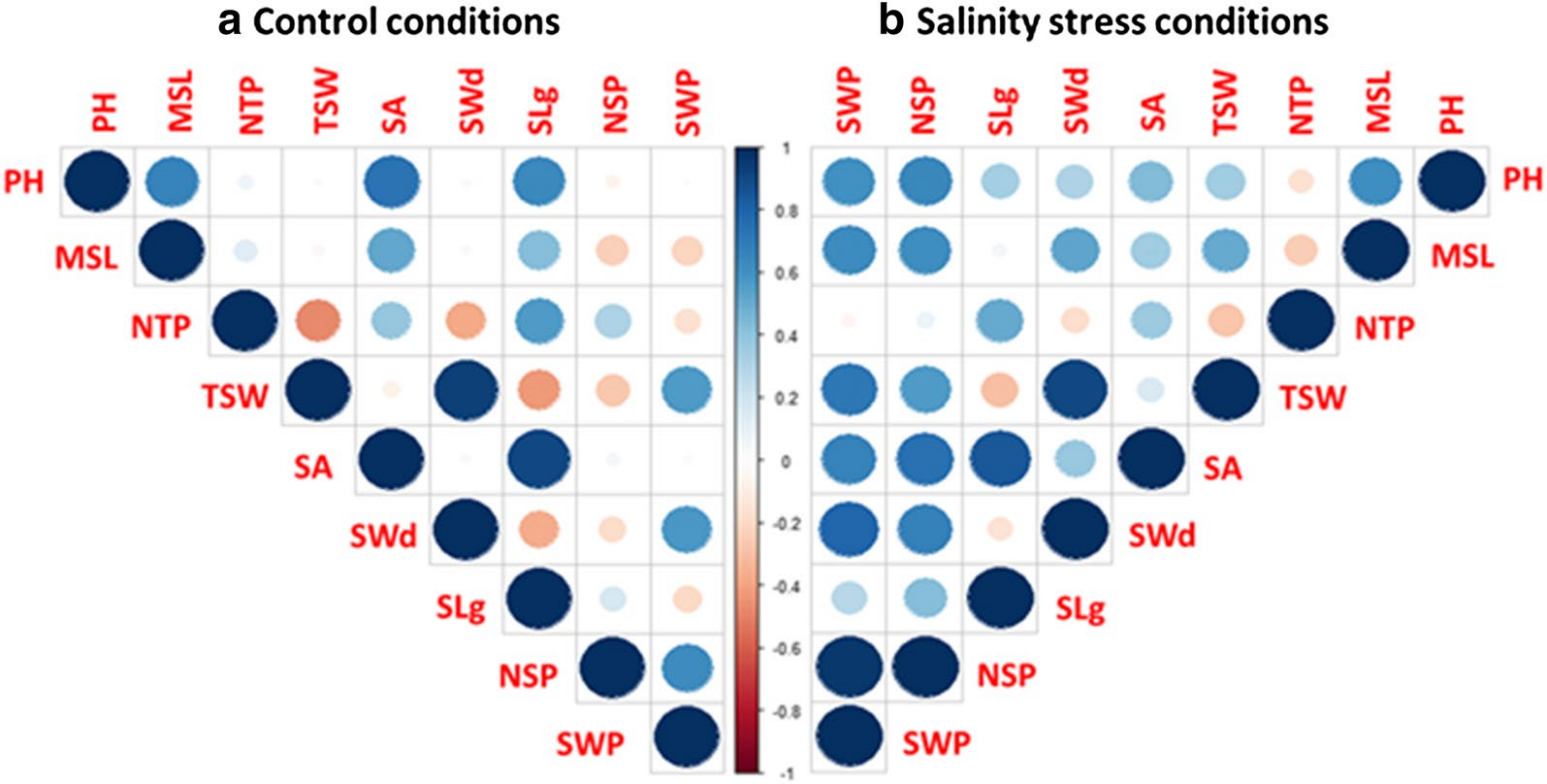
Correlation matrix heatmap among the phenotypic and yield traits: *PH* plant height, *MSL* main spike length, *NTP* number of tillers/plant, *TSW* thousand seeds weight, *SA* seed area, *SWd* seed width, *SLg* seed length, *NSP* number of seeds/plant, *SWP* seeds weight/plant. Shades of blue indicate increasing positive correlation coefficient; shades of red indicate increasing negative correlation coefficient (color figure online)

The effect of salt treatment on yield traits and their correlations of the five tested barley genotypes are shown in Additional file 3: Table S2 and Fig. 6. The data showed a clear reduction of all yield-related parameters investigated, i.e. spike length, seed length, seed width, seed area, number of seeds/plant, plant seeds weight and 1000-seed weight (TSW) (Additional file 3: Table S2). Under control condition, the five genotypes showed no significant differences in seed width. Values for the other parameters differed, however. Spike lenght, seed length, and seed area were significantly larger in the genotypes Ardhaoui and Rihane and distinctly smallest in the Lemsi genotype. Furthermore, number of seeds/plant was lowest in the genotypes Ardhaoui and Kounouz and highest in the genotypes Manel, Lemsi and Rihane while TSW was higher in the genotypes Kounouz and Manel and lower in Ardhaoui, Lemsi and Rihane (Additional file 3: Table S2). In all five genotypes, salinity conditions caused a significant reduction in the traits spike length, seeds/plant and seed weight while the traits seed length, seed width and seed area were not significantly affected. Number of seeds/plant and seeds weight was highest in the genotype Rihane and lowest in the genotype Lemsi. TSW was significantly reduced in the genotypes Ardhaoui and Lemsi, hardly affected in the genotypes Kounouz and Manel and significantly increased in the genotype Rihane. Overall, however, the negative effect of salinity treatment was strongest in the genotype Lemsi (Additional file 3: Table S2).

Under control conditions, data show different correlations between studied traits (Fig. 6a). Plant height was highly positive correlated with main spike length, seed area and seed length. Similarly, positive correlation was found for seed weight/plant which was highly correlated with TSW, seed width and number of seeds/plant. TSW was greatly correlated with seed width, the same for seed area and seed length, both correlations were positive. However, number of tillers/plant and seed length were negatively correlated with TSW and seed width. Under salinity stress treatment, new correlations between the traits comparing to control were detected (Fig. 6b). On the contrary of control conditions, plant height was positive correlated with number of seeds/plant and seed weight/plant, however, this positive correlation reduced with seed area and seed length. For main spike length, a negative correlation with number of seeds/plant and seeds weight/plant under control conditions turned into a positive correlation under salinity stress, a positive correlation with TSW was established. Number of seeds/plant became negatively correlated with TSW and seed width, the positive correlation between number of seeds/plant and seed weight/plant was more important under salt stress conditions than control. Regarding to seed area, two new positive correlations were generated under stress comparing to control.

## Discussion

The study for the first time describes the effect of salinity stress (EC level 12.5 ds/m) on spike developmental stages, spikelet primordia development and spike ABA concentrations in relation to yield in spring barley. The salinity caused reduction in the plant growth may be due to the negative effect of salt on many metabolic processes including protein nucleic acid and polyamine synthesis, transpiration, stomatal conductance, photosynthesis (Mittal and Dubey 1991; Reggiani et al. 1994; Netondo et al. 2004; Abbas et al. 2015; Bagues et al. 2018). Certain ions can restrict the absorption of water by plant roots (Mansour 1994; Ahmed et al. 2013), and/or induce an imbalance in phytohormone levels either through altered biosynthesis or a change in turn-over rates (Amzallage et al. 1992; Dunlap and Binzel 1996). Decreased plant height under the salinity might be due to the accumulation of salts in the cell wall limiting cell wall elasticity and cell elongation (Naseer et al. 2001; Taghipour and Salehi 2008; Colla et al. 2012) and resulting in stunted shoots (Aslam et al. 1993). Salinity stress also caused a significant reduction in number of tillers in all tested barley genotypes. These results concurred with the findings of Nicolas et al. (1994), Zhao et al. (2007), Shahzad et al. (2012) and Bagues et al. (2018).

**Fig. 7:**
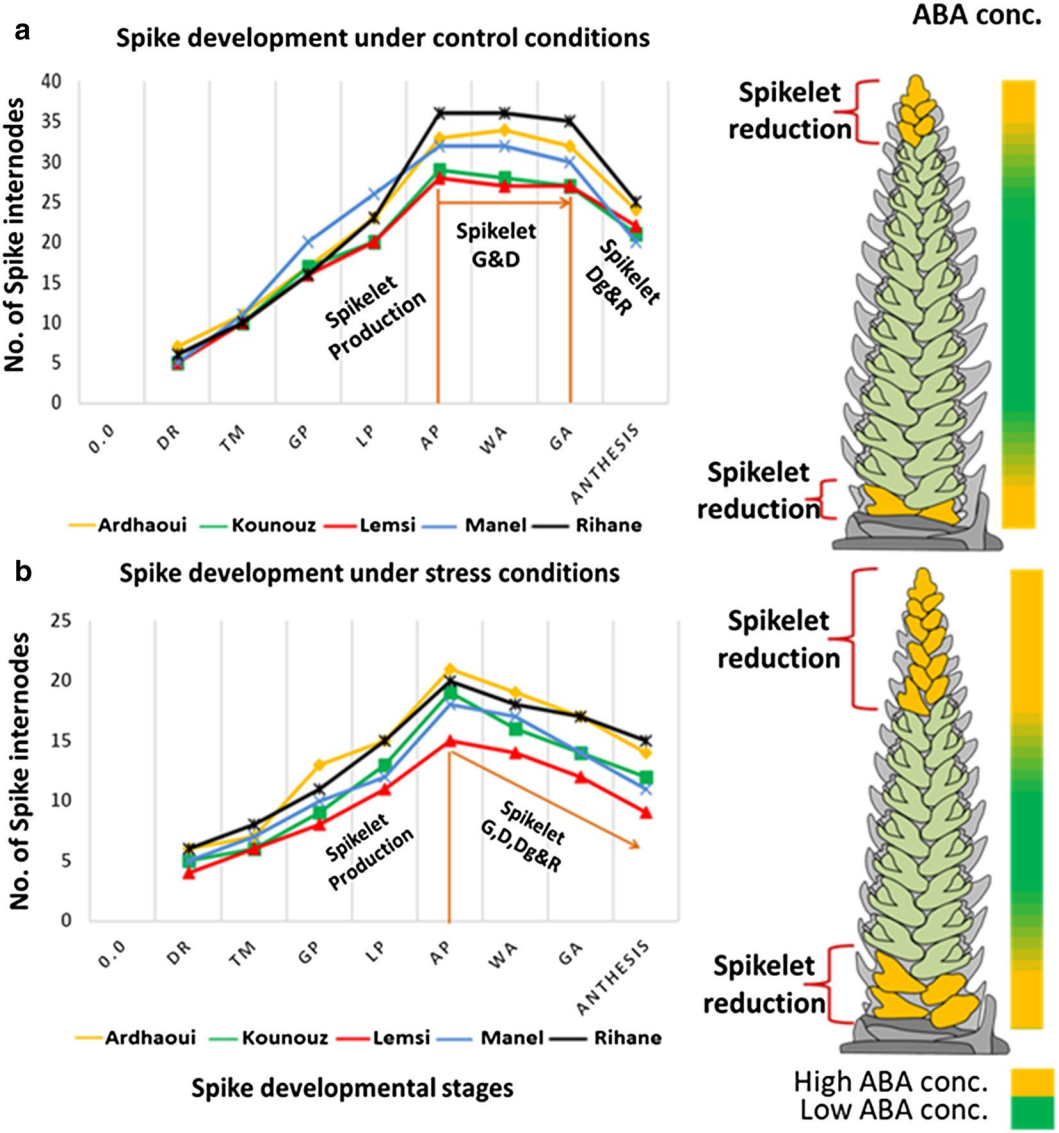
Barley spikelet production, growth, development, degradation and reduction and ABA concentration in the immature spike in the five tested barley genotypes under control (**a**) and salinity stress (**b**) conditions. *DR* double ridge, *TM* triple mound, *GP* glume primordia, *LP* lemma primordium, *AP* awn primordium, *WA* white anther, *GA* green anther, *G* growth, *D* development, *Dg* degradation, *R* reduction

Salinity treatment also had a direct effect on spike growth and spike development and through this an indirect effect on plant yield. We observed that under salinity treatment the spike developmental stages were shortened and degradation of spikelet primordia enhanced (Fig. 7). Although the underlying mechanisms are unclear, it is evident that under salinity stress the plant shortens its life cycle to rapidly produce a smaller number of seeds. Marschner (1971) reported that growing plants using saline water for irrigation affects physiological processes and reduces growth characters. Other studies have shown that the effect of salinity on yield components might be attributed to a rise in the osmotic pressure of rooting media which inhibits meristematic tissue activity and consequently leads to a reduction of the size and the number of cells per unit length (Thompson et al. 1997). Several authors have noted that salinity can inhibit plant growth by a range of mechanisms including osmotic effects which can restrict the absorption of water by plant roots, or direct ion toxicity and interference with the uptake of nutrients like K^+^ (Aldesuquy 1991; Abd El-Karim 1996; Zhu et al. 1998).

In our previous study (Youssef et al. 2017) we described the hormonal role in regulating floral organ patterning and phase duration during barley inflorescence and shoot development. Spike development is mainly influenced by phytohormones such as IAA, CK and gibberellins (Pearce et al. 2013). Atrophy and degradation of spikelet primordia in the apical and basal sections of the spike during green anther stage are additional proof of a hormonal role in spike and spikelet/floret development. The high concentrations of ABA in these sections at GA stage cause a local inhibition of floret development (Wang et al. 2000). In the central part of the spike, however, the lower concentration of ABA found in this study and higher concentration of gibberellins (our previous study, Youssef et al. 2017) allows the development of fertile flowers and grain setting (Fig. 7). Similar results were also reported by Wang et al. (1999), Cao et al. (2000), Youssef et al. (2017) and Youssef and Hansson (2019). This ABA dependent modification of the grain number at the apical and basal parts of the spike could be the mechanism by which the barley spike adapts its yield potential. Atkinson et al. (2013) reported on a negative correlation between the susceptibility to abiotic stress (salinity and drought) and ABA concentrations. In accordance to this we found highest ABA concentrations in the saline sensitive genotype Lemsi and lowest concentrations in the saline insensitive genotype Rihane (Fig. 5). In addition to the ABA effect, the spikelets/florets atrophy could be due to salt stress initially inducing osmotic stress and causing reduced water availability for the spikelets primordia, followed by ion toxicity due to nutrient imbalances in the cytosol causing spikelets/florets degradations and abortions.

In the present study, we also showed that salinity treatment adversely affects number of seeds/plant, weights of seeds/plant and TSW compared with the respective values in non-stressed control plants (Additional file 3: Table S2). The significant reduction in the grain yield under salt stress may be due to (i) an inhibition of tillering capacity (Fig. 2) causing a reduction in the number of spikes/plant as previously concluded by Al-Khafaf et al. (1990), Sakr et al. (2007) and Boussen et al. (2016), and (ii) enhanced degradation of the spikelet primordia through increasing of ABA concentrations in the apical and basal parts of the spikes (Figs. 4, 5, 7) reducing the number of seeds/spike. The detrimental and injurious effects of salinity on the growth productivity of barley shown here are in accordance with previous reports. Kumar et al. (1987) and Hank et al. (1989) showed that increasing salinity level in the irrigation water decreased growth and yield components of the plant. Holloway and Alston (1992) reported that salt stress decreased tillering, dry matter production and grain yield while Zeng and Shannon (2000) on rice and Sakr and El-Metwally (2009) on wheat, revealed that the reduction of tiller number/plant and spikelet number/panicle were the major causes of yield loss under salinity stress conditions. Sakr et al. (2004) suggested that the reduction in grain yield is largely due to a decrease in grain set which may be attributed to a decrease in the viability of pollen or in the receptivity of the stigmatic surface or both. Grattan et al. (2002) showed that salinity had negative impacts on number of spikes, tillers and spikelets per plant, floret sterility, individual grain size, and heading. We hypothesize that the reduction in grain yield of barley under salinity stress may be attributed to a diminished cell division and cell expansion in the spike, caused by altered concentrations of certain plant hormones like ABA. The ultimate result is a shortening of the spike developmental stages and a decreased production of developed spikelets and subsequent pollen grains. Hormones cross talking and their biological and genetic effects on spike and spikelet development appears a promising field for future work.

## Conclusion

Overall, salinity treatment reduced all yield-related parameters investigated and turned some of the correlations among them from positive to negative or vice versa. Investigations of ABA role in floral development and phase duration of barley spike showed that, ABA regulates the spikelet/floret abortion process affecting the yield potential under salinity and control conditions.

## Additional files

**Additional file 1: Fig. S1.** Plant growth and phenotype under control (a) and salinity stress (b) conditions of the five tested barley genotypes Ardhaoui (G1), Kounouz (G2), Lemsi (G3), Manel (G4) and Rihane (G5). C = control, T = Treatment (salinity).

**Additional file 2: Table S1.** Effect of salinity stress treatment (12.5 ds/m) on spike developmental stages for the five tested barley genotypes.

**Additional file 3: Table S2.** Mean values of yield traits, spike length (cm), seed length (mm), seed width (mm), seed area (mm^2^), number of seeds/plant, seeds weight/plant (g) and 1000-seed weight (TSW) (g) for the five tasted barley genotypes under control and salinity treatment conditions.

## Data Availability

Not applicable.

## Abbreviations

ABA: abscisic acid
GA: green anther
AP: awn primordium
*Vrs*: *six*-*rowed*-*spike*
IAA: auxin
CK: cytokinin
DR: double ridge
TM: triple mound
GP: glum primordia
SP: stamen primordium
LP: lemma primordium
WA: white anthers
HD: heading day
SEM: scanning electron microscopy
DAP: days after planting
TSW: 1000-seed weight
EC: electrical conductivity
