# Supplementary figures and images for: Spike developmental stages and ABA role in spikelet primordia abortion contribute to the final yield in barley (Hordeum vulgare L.)
Source: Bot Stud. 2019 Jul 10;60:13. doi: 10.1186/s40529-019-0261-2 (PMC6620232; doi:10.1186/s40529-019-0261-2)

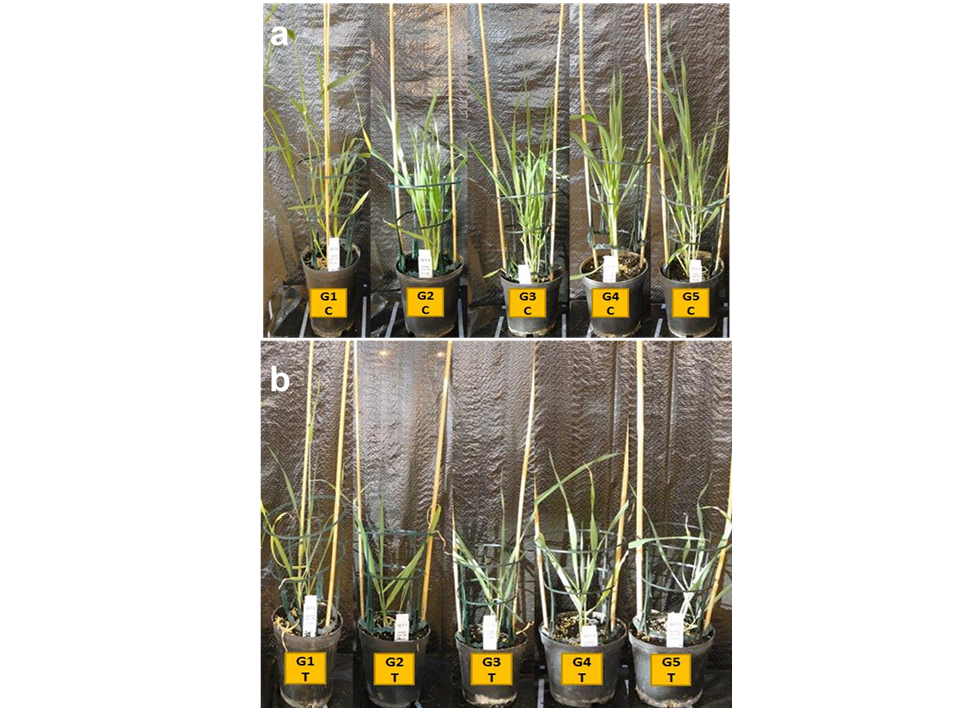

Supplement: Supplementary file 1 — Additional file 1: Fig. S1. Plant growth and phenotype under control (a) and salinity stress (b) conditions of the five tested barley genotypes Ardhaoui (G1), Kounouz (G2), Lemsi (G3), Manel (G4) and Rihane (G5). C = control, T = Treatment (salinity). [file 40529_2019_261_MOESM1_ESM.tif]
