# Supplementary material for: Spike developmental stages and ABA role in spikelet primordia abortion contribute to the final yield in barley (Hordeum vulgare L.)
Source: Bot Stud. 2019 Jul 10;60:13. doi: 10.1186/s40529-019-0261-2 (PMC6620232; doi:10.1186/s40529-019-0261-2)
Supplement: Supplementary file 2 — Additional file 2: Table S1. Effect of salinity stress treatment (12.5 ds/m) on spike developmental stages for the five tested barley genotypes. [file 40529_2019_261_MOESM2_ESM.docx]

Table S1 Effect of salinity stress treatment (12.5 ds/m) on spike developmental stages for the five tested barley genotypes.

|  |  | **Ardhaoui** | **Kounouz** | **Lemsi** | **Manel** | **Rihane** |
| --- | --- | --- | --- | --- | --- | --- |
|  |  | **(DAP)** | **(DAP)** | **(DAP)** | **(DAP)** | **(DAP)** |
| **DR** | **Control** | 13 | 12 | 11 | 12 | 13 |
|  | **Treatment** | 11 | 10 | 10 | 10 | 11 |
| **TM** | **Control** | 16 | 15 | 13 | 15 | 16 |
|  | **Treatment** | 14 | 13 | 12 | 13 | 14 |
| **GP** | **Control** | 17 | 17 | 15 | 17 | 18 |
|  | **Treatment** | 15 | 14 | 13 | 15 | 16 |
| **LP** | **Control** | 20 | 19 | 16 | 19 | 21 |
|  | **Treatment** | 18 | 16 | 15 | 18 | 20 |
| **AP** | **Control** | 26 | 24 | 22 | 25 | 26 |
|  | **Treatment** | 22 | 19 | 18 | 22 | 24 |
| **WA** | **Control** | 38 | 36 | 33 | 36 | 39 |
|  | **Treatment** | 30 | 28 | 24 | 31 | 35 |
| **GA** | **Control** | 48 | 45 | 42 | 44 | 51 |
|  | **Treatment** | 38 | 36 | 30 | 38 | 45 |
| **HD** | **Control** | 58 | 54 | 50 | 52 | 62 |
|  | **Treatment** | 45 | 43 | 33 | 44 | 54 |

DR = Double Ridge, TM = Triple Mound, GP = Glume Primordia, LP = Lemma primordium, AP = Awn primordium, WA = White Anther, GA = Green Anther, HD = Heading day. DAP = Days after planting
