# Supplementary material for: Spike developmental stages and ABA role in spikelet primordia abortion contribute to the final yield in barley (Hordeum vulgare L.)
Source: Bot Stud. 2019 Jul 10;60:13. doi: 10.1186/s40529-019-0261-2 (PMC6620232; doi:10.1186/s40529-019-0261-2)
Supplement: Supplementary file 3 — Additional file 3: Table S2. Mean values of yield traits, spike length (cm), seed length (mm), seed width (mm), seed area (mm2), number of seeds/plant, seeds weight/plant (g) and 1000-seed weight (TSW) (g) for the five tasted barley genotypes under control and salinity treatment conditions. [file 40529_2019_261_MOESM3_ESM.docx]

Table S2: Mean values of yield traits, spike length (cm), seed length (mm), seed width (mm), seed area (mm^2^), number of seeds/plant, seeds weight/plant (g) and 1000-seed weight (TSW) (g) for the five tasted barley genotypes under control and salinity treatment conditions.

|  |  | **Ardhaoui** | **Kounouz** | **Lemsi** | **Manel** | **Rihane** |
| --- | --- | --- | --- | --- | --- | --- |
| **Spike length (cm)** | **Control** | 5.58^**b^ | 4.66^*c^ | 4.20^**c^ | 4.74^**bc^ | 5.14^**bc^ |
|  | **Treatment** | 2.86^a^ | 3.23^a^ | 2.86^a^ | 3.13^a^ | 3.57^a^ |
| **Seed Length (mm)** | **Control** | 10.93^nsa^ | 8.69^*b^ | 8.43^*b^ | 9.13^*b^ | 10.54^nsa^ |
|  | **Treatment** | 10.61^c^ | 7.99^d^ | 7.76^d^ | 8.40^d^ | 10.27^c^ |
| **Seed Width (mm)** | **Control** | 3.07^*a^ | 3.21^nsab^ | 3.04^nsa^ | 3.33^nsb^ | 3.10^nsa^ |
|  | **Treatment** | 2.84^c^ | 3.05^d^ | 2.89^c^ | 3.17^d^ | 3.06^d^ |
| **seed Area (mm^2^** **)** | **Control** | 22.71^*a^ | 18.37^*c^ | 17.36^*c^ | 20.06^*b^ | 22.11^*a^ |
|  | **Treatment** | 20.41^d^ | 16.53^e^ | 15.62^e^ | 18.05^f^ | 16.52^e^ |
| **N. Seeds/plant** | **Control** | 89.67^**a^ | 87.20^**a^ | 101.20^**ab^ | 100.20^**ab^ | 117.11^**b^ |
|  | **Treatment** | 14.90^c^ | 14.71^c^ | 6.10^d^ | 16.14^c^ | 36.29^e^ |
| **Seeds Weight (g)** | **Control** | 2.52^**a^ | 2.87^**a^ | 2.79^**a^ | 2.97^**a^ | 3.76^**b^ |
|  | **Treatment** | 0.33^c^ | 0.45^c^ | 0.14^d^ | 0.55^c^ | 1.29^d^ |
| **TSW (g)** | **Control** | 29.40^**f^ | 33.00^*e^ | 27.76^**f^ | 37.34^**e^ | 35.22^nse^ |
|  | **Treatment** | 18.19^a^ | 28.36^c^ | 15.92^a^ | 24.61^b^ | 31.18^d^ |

Red stars and non-significant ‘ns’ show the significant differences between the control and salinity treatment for each genotype. Black letters show the significant differences among the genotypes and genotypes-treatment interactions. Significant differences has been calculated using R 3.5.3.
